# EGb761 improves the cognitive function of elderly db/db^−/−^ diabetic mice by regulating the beclin-1 and NF-κB signaling pathways

**DOI:** 10.1007/s11011-018-0295-2

**Published:** 2018-09-05

**Authors:** Zhu-Fei Guan, Xiao-Ming Zhang, Ying-Hong Tao, Yu Zhang, Yan-Yan Huang, Gang Chen, Wei-Jun Tang, Gang Ji, Qi-Lin Guo, Ming Liu, Qian Zhang, Na-Na Wang, Zhong-Yu Yu, Hao-Yang, Guo-Feng Wu, Zhou-Ping Tang, Zun-Guo Du, Xi-Liang Shang, Ying-Chao Liu, Guang-Hai Mei, Jing-Chun Guo, Hou-Guang Zhou

**Affiliations:** 1 Department of Geriatrics, National Clinical Research Center for Aging and Medicine, Huashan Hospital, Fudan University, Shanghai, 200040 China; 2 State Key Laboratory of Medical Neurobiology, Department of Neurobiology, School of Basic Medical Neurobiology, Department of Neurobiology School of Basic Medical Science, Shanghai Medical College, Fudan University, Shanghai, 200032 China; 3 Department of Medical Examination Center, Huashan Hospital, Fudan University, Shanghai, 200040 China; 4 Department of Radiology, Huashan Hospital, Fudan University, Shanghai, 200040 China; 5 Department of Emergency Neurology, Guiyang Medical University, Guiyang, 550004 China; 6 Department of Neurology, Tongji Hospital, Huazhong University of Science and Technology, Wuhan, 430000 China; 7 Department of Pathology, Huashan Hospital, Fudan University, Shanghai, 200040 China; 8 Department of Sport Medicine, Huashan Hospital, Fudan University, Shanghai, 200040 China; 9 Department of Neurosurgery, Provincial Hospital Affiliated to Shandong University, Jinan, 250021 China; 10 Department of Neurosurgery, Huashan Hospital, Fudan University, Shanghai, 200040 China

**Keywords:** EGb761, Beclin-1, NF-κB protein, LC3-II/I, Autophagy, Cognitive function

## Abstract

To assess whether EGb761 could protect elderly diabetic mice with cognitive disorders and explore the role of beclin-1-mediated autophagy in these protective effects. Two-month-old male db/db^−/−^ mice and wild-type C57/BL6 mice were randomly divided into six groups: db/db^−/−^ control, db/db^−/−^ 50 mg, db/db^−/−^ 100 mg, wild-type (WT) control, WT 50 mg, and WT 100 mg. EGb761 (50 mg/kg or 100 mg/kg of bodyweight) was given by gavage once a day for 1 month from the age of 6 months. Y-maze and social choice tests were performed at 8th months. The blood pressure was measured. The imaging changes in the brain were measured using magnetic resonance imaging (MRI). The expression and distribution of beclin-1, LC3, and NF-κB were detected using immunohistochemistry staining and western blotting. Ultrastructure alterations in the hippocampus were observed using transmission electron microscopy. Compared with WT mice, the learning ability, memory and overall cognitive function of db/db^−/−^ mice decreased (*P* < 0.05), and EGb761 could significantly improve the learning and memory function of db/db^−/−^ mice (*P* < 0.05). EGb761 significantly improved systolic blood pressure in db/db^−/−^ mice (*P* < 0.01). In addition, fMRI-bold showed a decline in the hippocampus of mice in the db/db^−/−^ group compared with WT. EGb761 could improve these above changes. Immunohistochemistry staining and western blotting confirmed that EGb761 significantly increased beclin-1 and reduced LC3-II/I levels in the brains of db/db^−/−^ mice (*P* < 0.05). NF-κB levels were obviously higher in the db/db^−/−^ group than that in the WT group, and EGb761 significantly reduced NF-κB levels in db/db^−/−^ mice (*P* < 0.05). There was a trend of increased autophagosomes in db/db^−/−^ mice, but EGb761 did not change obviously the number of autophagosomes. Compared with normal aged WT mice, aging db/db^−/−^ mice had more common complications of cerebral small vessel disease and cognitive dysfunction. EGb761 could significantly improve the cognitive function of aging db/db^−/−^ mice via a mechanism that may involve the regulation of beclin-1, LC3, and NF-κB.

## Introduction

Type 2 diabetes mellitus (T2DM) and cerebral small vessel disease (CSVD) are two of the most common diseases in the elderly population. According to the statistical results published by the International Diabetes Federation, the number of T2DM patients in China reached 96 million in 2014, and the morbidity was the highest in the world, higher than India and the United States (Whiting et al. 2011). Moreover, T2DM is an independent risk factor for cerebrovascular disease (Zhou et al. 2009). Since CSVD induces collateral circulation dysfunction in the brain, cognitive disorders are one of the common complications in patients with T2DM (Chan et al. 2013; Imamine et al. 2011). Patients with T2DM are also more likely to suffer from memory disorders (Janson et al. 2004), and T2DM-associated cognitive dysfunction is mostly accompanied by changes in ultrastructure and neurochemicals in the brain (Miles and Root 1924; Biessels et al. 1994). However, there is a lack of clinical therapies which are effective against the T2DM cognitive disorders.

Autophagy is a physiological process in which lysosomal enzymes remove damaged organelles or misfolded proteins; it plays an important role in the normal functioning of cells in the body (Bernales et al. 2007; Rodriguez-Enriquez et al. 2006). The cells must remove denatured macromolecules or destroyed cytoplasmic organoids continuously to maintain the appropriate function. During stressful situations, autophagy is an important mechanism which promotes cell survival. However, some evidence has suggested that autophagy could accelerate cell death (Wang and Miao 2012; Wei et al. 2012). Recent studies revealed that autophagy likely participated in the pathologic development of T2DM (Singh et al. 2009; Donohue 2009; Gonzalez et al. 2011). In addition, accumulating evidence has demonstrated that autophagy is closely related to cognitive disorders in T2DM (Taubes 2003; Reagan 2012). Therefore, drugs that intervene with autophagy are expected to be clinical candidates.

EGb761, a *Ginkgo biloba* extract including 24% flavonoids and 6% terpene lactones, has been used to improve cardiovascular and peripheral vascular insufficiency, to protect against neurological disorders like ischemic injury, and to treat cerebral disorders including cognitive decline and memory impairment (Defeudis 2002). Since EGb761 could alleviate dementia and protect neurons, we have assessed whether EGb761 could modulate cellular autophagy and reduce the risks of T2DM-induced cognitive degeneration.

In the present study, elderly db/db^−/−^ T2DM mice were used to investigate the effects of EGb761 on cognition and observe changes in the brain ultrastructure and beclin-1 protein levels which was autophagy-related. The results demonstrated that EGb761 could significantly improve the cognitive function of elderly T2DM mice via a mechanism which may be related to the modulation on beclin-1 and NF-κB signaling.

## Materials and methods

### Animal grouping

All mice were purchased from the Model Animal Research Center of Nanjing University. EGb761 (40 mg/ml in nose drops) was produced by Weimar Shu Pei Company, Germany. Two-month-old male db/db^−/−^ mice and wild-type C57 BL6/J (WT) mice were randomly divided into six groups: db/db^−/−^ control, db/db^−/−^ 50 mg, db/db^−/−^ 100 mg, wild-type (WT) control, WT 50 mg, and WT 100 mg. When the mice were 6 months old, EGb761 (50 mg/kg or 100 mg/kg of bodyweight) was given by gavage once a day for 1 month.

### Y-maze test

The Y-maze is a simple two-trial recognition test for evaluating spatial recognition memory. Mice were placed in one arm, and the sequence and number of arm entries were recorded for each mouse over a 10-min period, as described previously (Kim et al. 2007). The percentage of triads in which all three arms were represented, i.e., ABC, CAB, or BCA but not BAB, was recorded as an alternation to estimate short-term memory. The maze arms were cleaned thoroughly and sprayed with water between tests to remove residual odors. The alternation score for each mouse was defined as the ratio of the actual number of alternation to the possible number of alternation multiplied by 100 as the following equation: ([number of alternations] / [total arm entries – 2]) × 100. The total number of arm entries acted as an indicator of locomotor activity.

### Social choice test

Social choice tests were conducted as described previously (Syed et al. 2015). Each test consisted of two sessions: a social interaction session and a social novelty preference session. Each session lasted for 10 min with a 20-min inter-session interval. At the start of the experiment, the animal was placed in the center of a square box (40 × 40 × 40 cm) and was given 5 min to acclimatize to the novel environment. After their acclimatization, the social interaction session started by the introduction of a small wire cage carrying a mouse of the same weight, age, and strain (C1); another empty cage of the same dimensions was placed diagonally to the first cage. After completing the first session, the animal was returned to its home cage. In the social novelty preference session, a new mouse (C2) was introduced into the empty cage and the familiar mouse (C1) remained in the same cage as in the previous session. In both sessions, the interaction time was recorded when the test mouse touched the cage or physically contacted the C1 or C2 mouse.

### Blood pressure detection

Blood pressure (BP) was measured using the BP machine (Visitech systems BP-2000, USA) (Guan et al. 2017). Mice were allowed to acclimate to the pressure cuff on their tail for five minutes, and they were placed on a warming platform before recording BP measures. The BP measures consisted of 25 cycles of diastolic/systolic measures, with a 20 s rest period between cycles. Following the data collection, the mice were immediately released back into their homecages. The rodent restraints, cuffs, and warming platform were cleaned between subjects. The BP measures were obtained at the same time each day to account for the possible influence of circadian rhythms.

### MRI scanning

Each animal was anesthetized using isoflurane and fixed on the scanning bed of an MRI (BioSpec 70/30 USR, Bruker, Switzerland). Rapid acquisition with relaxation enhancement (RARE) sequences were used to obtain anatomic images of the brain, and data regarding brain functions were collected using gradient-echo. Statistical parametric mapping (SPM), dealing with MRI-bolding, and the resting state fMRI data analysis tools were used to filter and attenuate the line of the reserved brain tissues; the results were then Fourier-transformed to obtain the low-frequency fluctuations (LFF) power within 0.0–0.1 Hz and describe the spectrum. Then, the amplitude of low-frequency fluctuations (ALFF) power was calculated within 0.01–0.1 Hz and the results were drawn into a chart after being transferred. The functional connections between the hippocampus and other areas were analyzed to consult the stereotaxic atlas and gain a functional linkage map of the brain. Finally, the data were analyzed statistically and the results were obtained to confirm the site of the signal changes. A Bruker 3.1 7 T superconduct MRI system was used and the inner diameter of the coil was 47 mm. The scanning parameters were as follows: echo time, 20 s; repetition time, 2000 ms; band width, 333,333.3 Hz; number of plies, 16; depth of plies, 0.8 mm; image size, 64; and scanning field, 21,600–15,000 mm.

### Immunohistochemistry

Brain tissue sections were dewaxed by placing in xylene twice for 15 min each, absolute ethanol twice for 5 min each, 85% ethanol for 5 min, 75% ethanol for 5 min, and finally in water. Then, the sections were repaired using EDTA antigen retrieval buffer and incubated with rabbit polyclonal antibodies against beclin-1 (1:500; Santa Cruz Biotechnology, Santa Cruz, CA, USA), LC3 (1:200; Cell Signaling Technology, Danvers, MA, USA) in PBS containing 3% BSA overnight at 4 °C. The samples were then incubated with anti-rabbit antibodies for 45 min at room temperature. Next, the sections were colored with DAB and the nuclei were stained with hematoxylin for ~5 min, washed with water, washed with 1% hydrochloric-alcohol solution, washed with water, colored blue with ammonia, and then washed with water. Finally, the sections were dehydrated sequentially with 75% ethanol for 5 min, 85% ethanol for 5 min, ethyl alcohol twice for 5 min each, and xylene for 6 min. They were then covered on slides for image analysis. During the immunohistochemistry image analysis, the nuclei that were stained with hematoxylin were blue and those colored by DAB were brown.

### Western blotting

Western blotting was performed as described previously (Mlyniec et al. 2014). The hippocampi were homogenized using RIPA buffer (20 mg of tissue with 200 μl of RIPA). The tissue lysates were centrifuged at 12,000 rpm for 5 min and the supernatants were collected to determine the protein concentrations using a bicinchoninic acid protein assay (Beyotime Institute of Biotechnology, Shanghai, China). The membranes were re-probed with an antibody specific to GAPDH as an internal control. The specific primary antibodies used included rabbit polyclonal antibodies against beclin-1 (1:500; Santa Cruz), LC3 (1:200; Cell Signaling Technology), NF-κB (1:200; Santa Cruz), and GAPDH (1:2000, Boster, Shanghai, China). Finally, the X-ray films were developed and fixed in a dark room.

### Transmission electron microscopy

Transmission electron microscopy (TEM, PHILIPS CM-120, Netherlands) was performed as described previously (Guan et al. 2016). Brain tissues were perfused with 2.5% glutaraldehyde perfusate (25% glutaraldehyde and 0.2 M phosphate buffer with 3mMMgCl2, pH 7.4), followed by fixation with 2.5% glutaraldehyde as previously described (Gonzales et al. 2013). And then concentional of fixation solution, dehydrated, embedded in paraffin, sliced and 3% uranyl acetate and lead citrate double staining. The CM-120 PHILIPS was observed and photographed under transmission electron microscope.

### Statistical analysis

All statistical analyses were performed using PRISM software (GraphPad, La Jolla, CA, USA). All of the data are presented as means ± SEM. The data were evaluated with ANOVA and bonferroni correction was used for multiple comparison. *P* values less than 0.05 were considered significant.

## Results

### EGb761 had a potential to improve the learning-memory capacity of elderly diabetic mice

**Fig. 1:**
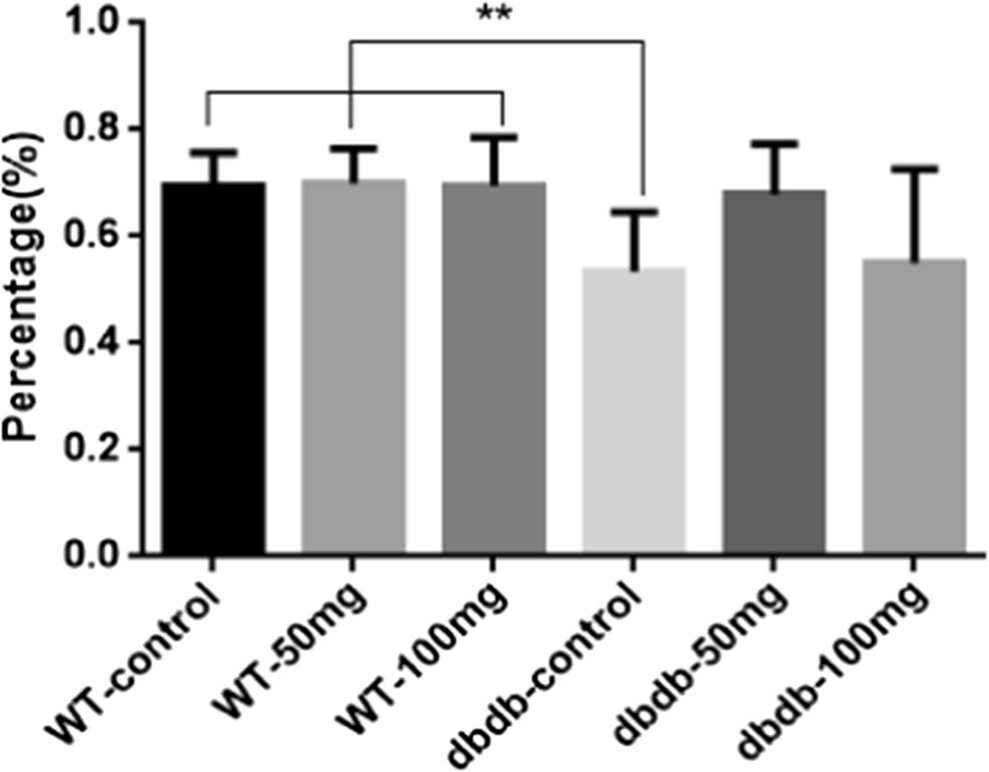
**The results of the Y-maze tests.** The alternation percentage was significantly lower in the db/db^**−/−**^ control group than that in the three WT groups (**, *P*<0.01, F = 7.330). The percentage of lower dose of EGb761 (50 mg/kg) had a rising trend compared with db/db^**−/−**^-control group, which implied a better memory ability. Alternation percentage = actual alternation / maximum alternation. *n* = 8 for WT control, WT 50 mg, WT 100 mg and db/db^**−/−**^ control; *n* = 6 for db/db^**−/−**^ 50 mg; *n* = 7 for db/db^**−/−**^ 100 mg

The results of the Y-maze test showed that the alternation percentage in the db/db^−/−^ control group was significantly lower than that in the three WT groups(*P*<0.01, F = 7.330), indicated that the learning and memory abilities were reduced in db/db^−/−^-control mice (*P* < 0.05 vs. all three WT groups). The lower dose of EGb761 (50 mg/kg) had a obvious trend to increase the alternation percentage, while no statistical significance when compared with db/db^−/−^-control group, which implied lower dose of EGb761 had a potential protective effect to the learning and memory function of mice in the db/db^−/−^ 50 mg group. The higher dose of EGb761 (100 mg/kg) did not induce obvious effect compared with that in db/db^−/−^ control mice (Fig. 1). Alternation percentage = actual alternation / maximum alternation. *n* = 8 for WT control, WT 50 mg, WT 100 mg and db/db^−/−^ control; *n* = 6 for db/db^−/−^ 50 mg; *n* = 7 for db/db^−/−^ 100 mg.

### EGb761 did not improve the social novelty preferences of elderly diabetic mice

**Fig. 2:**
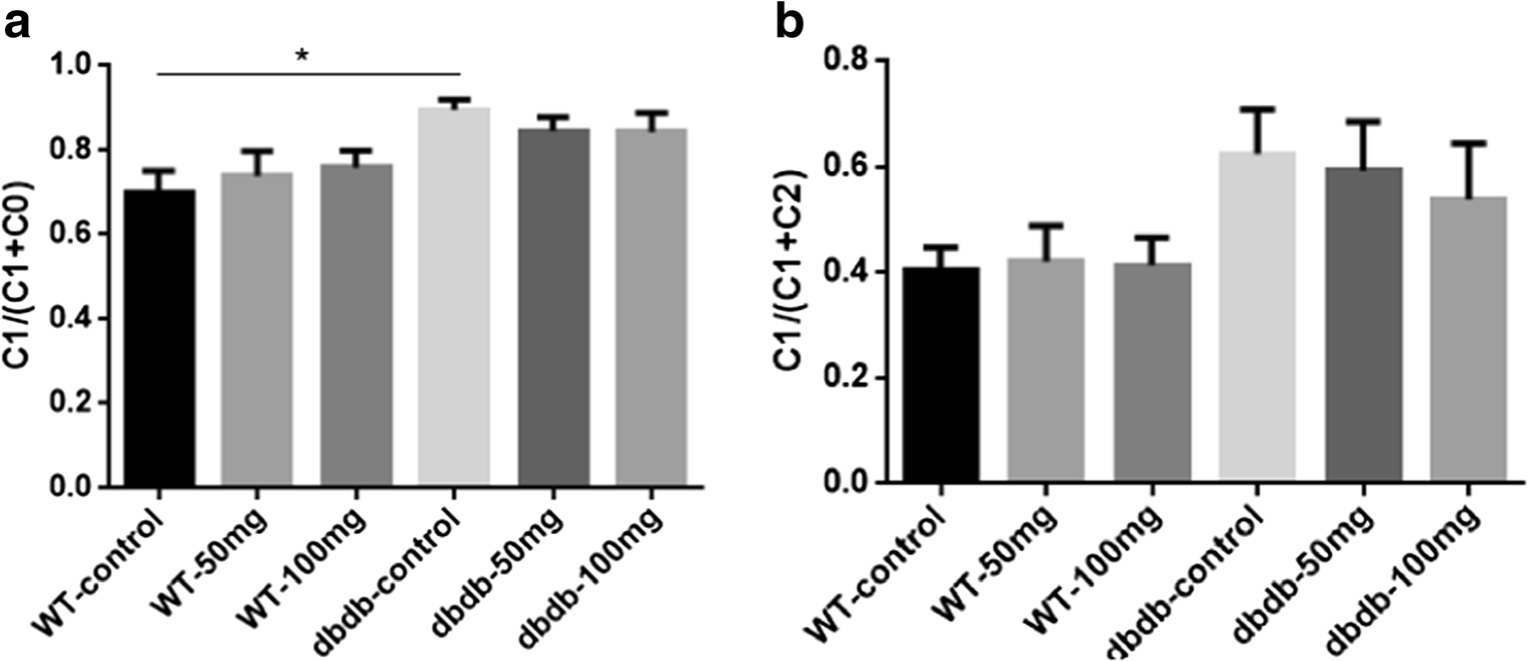
**Effects of EGb761 on sociability (a) and social memory (b). a** During sociability testing, the times spent in the compartment containing C1 was more than C0. In addition, db/db^**−/−**^ control mice spent significantly longer pursuing C1 than the other five groups, and the difference compared with WT control was significant (*, *P* < 0.05, F = 3.013). A: C1 = stranger 1, C0 = empty cage. **b** During the social memory testing, compared with three WT groups, the mice in dbdb^**−/−**^ control and dbdb^**−/−**^ 50 mg groups spent more time pursuing C1, and there were also a downward trend of db/db^**−/−**^ 50 mg and db/db^**−/−**^ 100 mg groups (*P = 0.1814*, F = 1.606). B: C1 = stranger 1, C2 = stranger 2. n = 8 for WT control, WT 50 mg, WT 100 mg, db/db^**−/−**^ control, and db/db^**−/−**^ 100 mg; n = 6 for db/db^**−/−**^ 50 mg

In the adaptation and training stage, all mice had no interest in the two-sided empty cages, and there was no difference in the time taken to exploring the cages. As the results showed, the ratios of C1/(C1 + C0) were all more than 0.5, and it suggested that in the social action stages, both db/db^−/−^ and WT mice developed preferences for the novel mice (C1). The db/db^−/−^ control mice pursued C1 mice for longer than those in the other five groups, especially compared with WT control group (*P*<0.05), suggesting that diabetic mice like social contact more. EGb761 could reduce the preference for novel subjects in db/db^−/−^ mice and reverse the social preference back to the levels of WT mice. In the social novelty preference testing stage, we could see that the C1/(C1 + C2) in WT groups were less than 0.5, and it suggested that WT mice preferred to stay with the novel mice (C2) longer than with the C1 mice; however, db/db^−/−^ mice expressed stronger social preferences for C1 mice rather than C2 (Fig. 2b), but there were no differences in all groups. These results suggested that db/db^−/−^ mice had difficulty with novelty recognition. EGb761 treatment did not change the preference trends in either WT or db/db^−/−^ mice.

### EGb761 could significantly improve systolic blood pressure of db/db^−/−^ mice

**Fig. 3:**
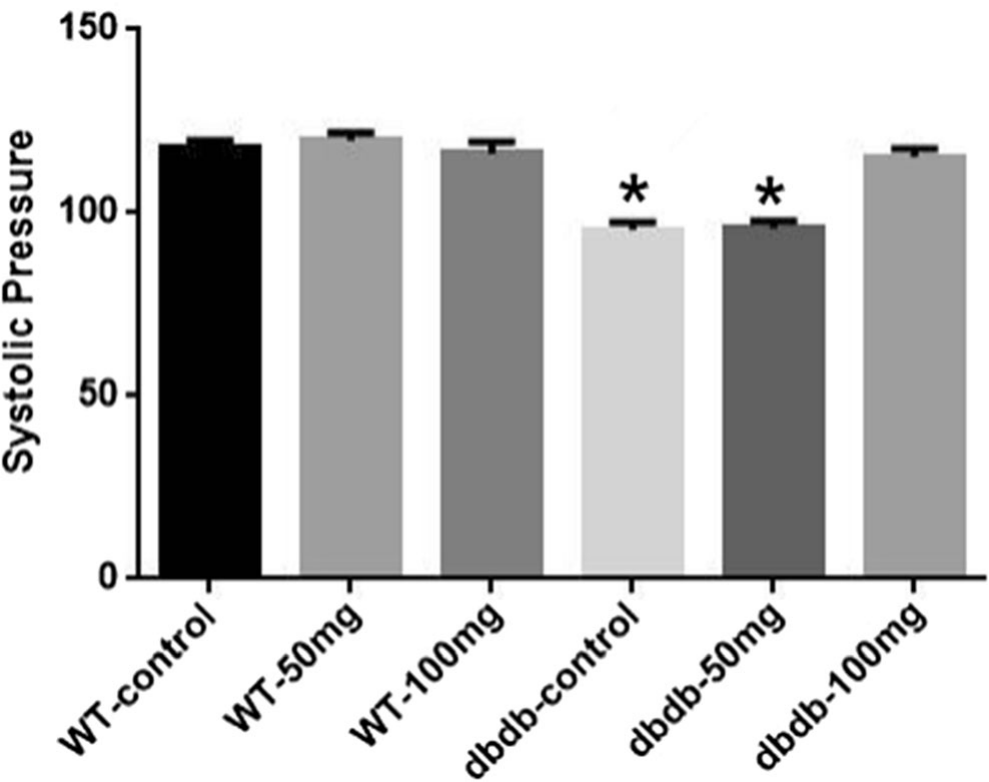
**Effect of EGb761 on syscolic pressure in mice.** The syscolic pressure of db/db^**−/−**^ control and db/db^**−/−**^ 50 mg groups were decreased compared with WT groups and db/db^**−/−**^ 100 mg group (*, *P*<0.05, F = 26.39). And the systolic pressure of db/db^**−/−**^ 100 mg group mice was moderate and recovered to the normal level almostly. n = 7

Compared with WT mice, the syscolic pressure of db/db^−/−^ control and db/db^−/−^ 50 mg groups tended to decrease compared with WT groups (*P*<0.05). And the systolic pressure of db/db^−/−^ 100 mg group mice was moderate and recovered to the normal level almostly. But the pressure of two WT groups with EGb761 were not raised. But there was no significant difference in diastolic blood pressure among these groups. These results suggested that EGb761 only improved abnormal blood pressure in db/db^−/−^ mice but did not affect normal blood pressure in WT mice (Fig. 3).

### EGb761 could partially improve nervous activation in the brains of elderly db/db^−/−^ mice

**Fig. 4:**
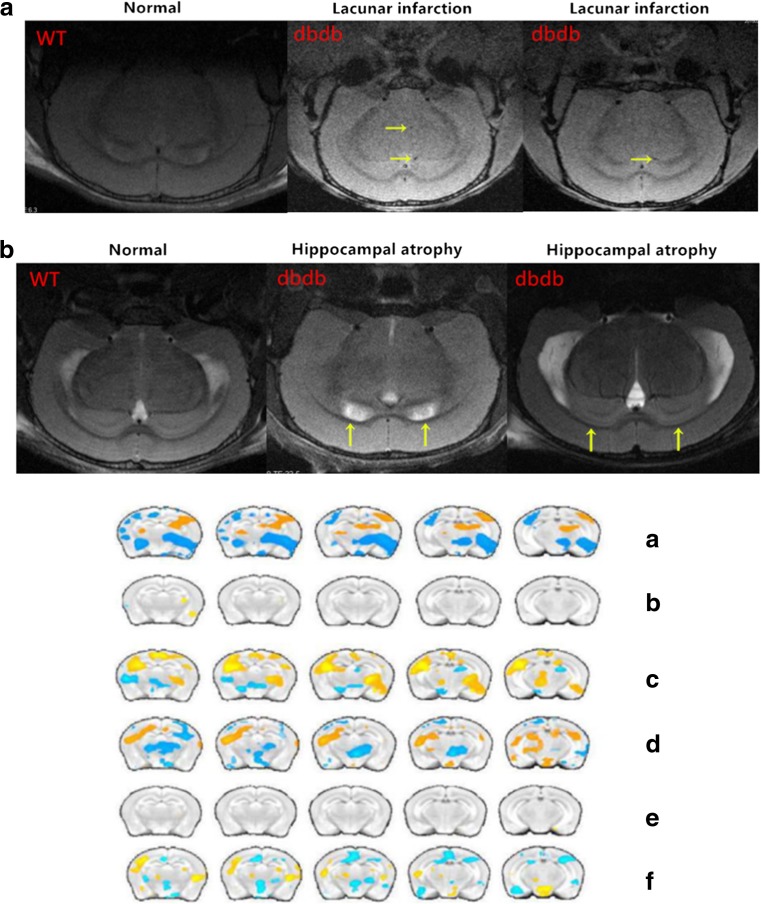
**The Magnetic resonance images.** A: The results showed that cerebral small vessel disease were present in the brains of db/db^**−/−**^ mice; B:The results showed that hippocampi atrophy and ventricular enlargement were present in the db/db^**−/−**^ mice. The fMRI-bold results of the mice in each group. A: The ALFF values of the hippocampus of mice in the db/db^**−/−**^/WT control groups. B: The ALFF values of the hippocampus of mice in the WT 50 mg/WT control group. C: The ALFF values of the hippocampus of mice in the db/db^**−/−**^ 50 mg/db/db^**−/−**^ control group. D: Functional connectivity map of WT control mice and db/db^**−/−**^ control mice using the hippocampus as the region of interest. E: Functional connectivity map of WT 50 mg mice and WT control mice using the hippocampus as the region of interest. F: Functional connectivity map of db/db^**−/−**^ 50 mg mice and db/db^**−/−**^ control mice using the hippocampus as the region of interest. *n* = 3

To investigate whether EGb761 could modulate neuronal activity in elderly db/db^−/−^ mice, fMRI-bold was performed in the brain. The MRI results demonstrated that most db/db^−/−^ mice had multiple lacunae infarct lesions in the brain, which belonging to CSVD, and some even had hippocampus atrophy and ventricular enlargement (Fig. 4). In addition, the intensity of the hippocampal ALFF signal was weaker in db/db^−/−^ mice than in WT mice, indicating that the nervous function of db/db^−/−^ mice had declined, similarly to the connections between the hippocampus and its peripheral region. Although 50 mg/kg EGb761 enhanced the connections in db/db^−/−^ mice, it had no effect on WT mice (Fig. 6).

**Fig. 5:**
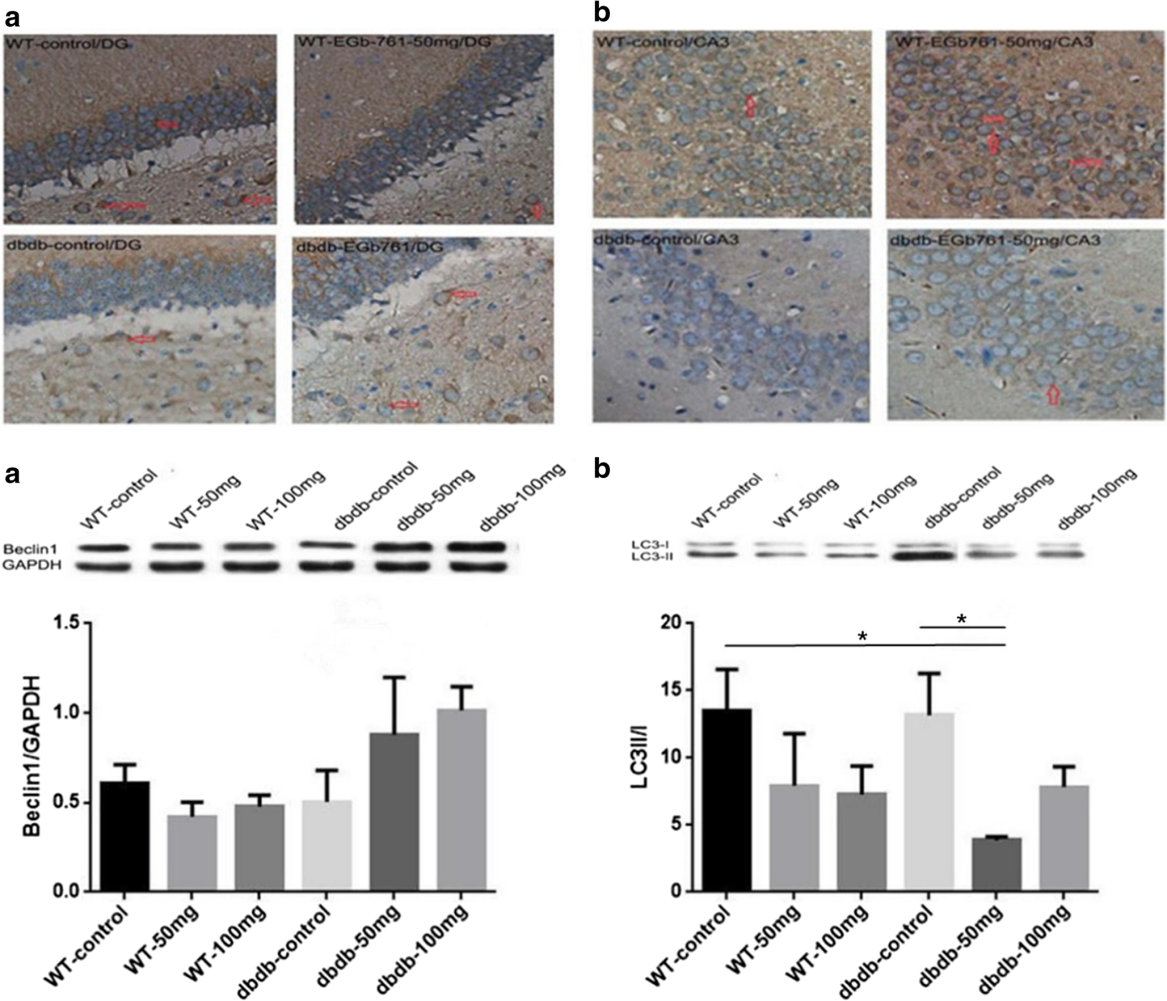
**Immunohistochemistry staining for LC3 (a) and beclin-1 (b) in the DG and CA3 areas of the hippocampus.** A: Representative images of LC3 staining. B: Representative images of beclin-1 staining. Scale bar = 1:20. Beclin-1 and LC3 in levels in the hippocampus were measured by western blotting. The graphs show the relative density of beclin-1 to GAPDH (**a**) and the LC3-II/I ratio (**b**). There were trend of growth with beclin-1 expression in the db/db^**−/−**^ 50 mg and 100 mg group compared with that in the db/db^**−/−**^ control and three WT groups, but without difference. The LC3-II/I ratio was decreased in the db/db^**−/−**^ 50 mg and db/db^**−/−**^ 100 mg groups; the difference of db/db^**−/−**^ 50 mg group compared with WT control group was significant (*, *P* < 0.05, F = 3.362); also the difference between db/db^**−/−**^ 50 mg group and db/db^**−/−**^ control group was significant (*, *P* < 0.05, F = 3.362). n = 8 for WT 100 mg and db/db^**−/−**^ 100 mg; n = 7 for WT 50 mg and db/db^**−/−**^ control; *n* = 5 for WT control and db/db^**−/−**^ 50 mg

**Fig. 6:**
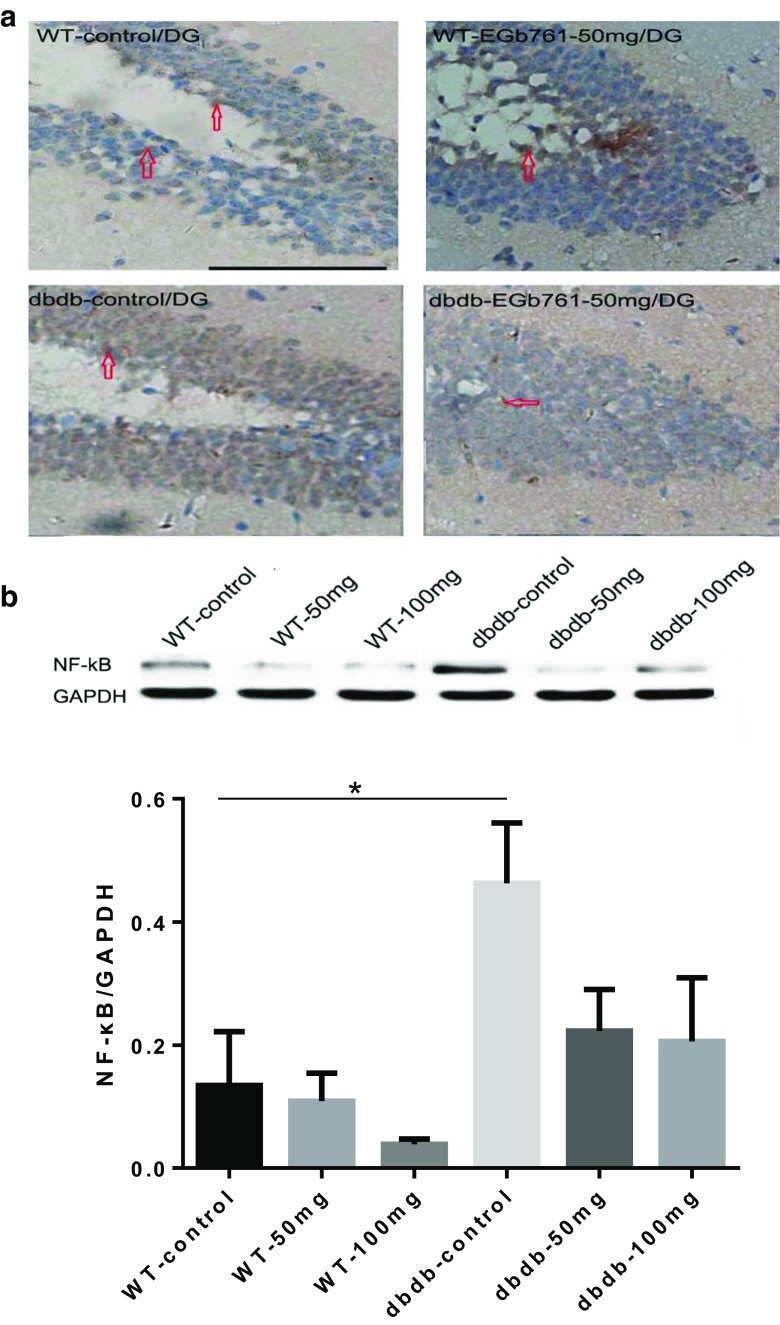
**NF-κB immunohistochemistry staining. a** Representative immunohistochemistry images of NF-κB staining. EGb761 reduced the expression of NF-κB protein in db/db^**−/−**^ mice, but did not change NF-κB levels in WT mice. **b** The levels of NF-κB in the hippocampus were measured by western blotting. The graphs showed the relative levels of NF-κB to GAPDH. NF-κB expression was increased significantly in db/db^**−/−**^ control compared with WT control (*, *P* < 0.05, F = 9.088). EGb761 reduced NF-κB levels in the db/db^**−/−**^ groups without difference. n = 8 for WT 100 mg; n = 7 for WT 50 mg, db/db^**−/−**^ control, and db/db^**−/−**^ 100 mg; n = 5 for WT control and db/db^**−/−**^ 50 mg

### EGb761 promotes the expression of beclin-1 protein and reduces the LC3-II to LC3-I ratio in the hippocampus of elderly diabetic mice

The protein expression of LC3 and beclin-1 was assessed using immunohistochemistry and western blotting. As shown in Fig. 4, the expression of both proteins was lower in the hippocampus of db/db^−/−^ mice than in WT mice. However, db/db^−/−^ mice fed with EGb761 expressed more LC3 and beclin-1 than those that did not receive EGb761. The western blotting results confirmed that beclin-1 levels had a tendency to rise in the db/db^−/−^ groups with EGb761 compared with those without EGb761. Treatment with 50 mg/kg, but not 100 mg/kg, EGb761 could significantly reduce the LC3-II/I ratio compared with that in the db/db^−/−^ control group (*P* < 0.05; Fig. 6).

### EGb761 reduced the expression of inflammatory factors in the hippocampal area of db/db^−/−^ mice

The expression of NF-κB protein was increased in the hippocampal area of db/db^−/−^ mice compared with that in the WT (*P*<0.05). However, EGB761 reduced NF-κB levels in db/db^−/−^ mice. Meanwhile, the western blotting results revealed the same outcome; the NF-κB protein content was increased in db/db^−/−^ mice, but not in WT. However, there were no dose-dependent effects (Fig. 6).

### Autophagy was increased in aging db/db^−/−^ mice compared with WT, but not significantly

**Fig. 7:**
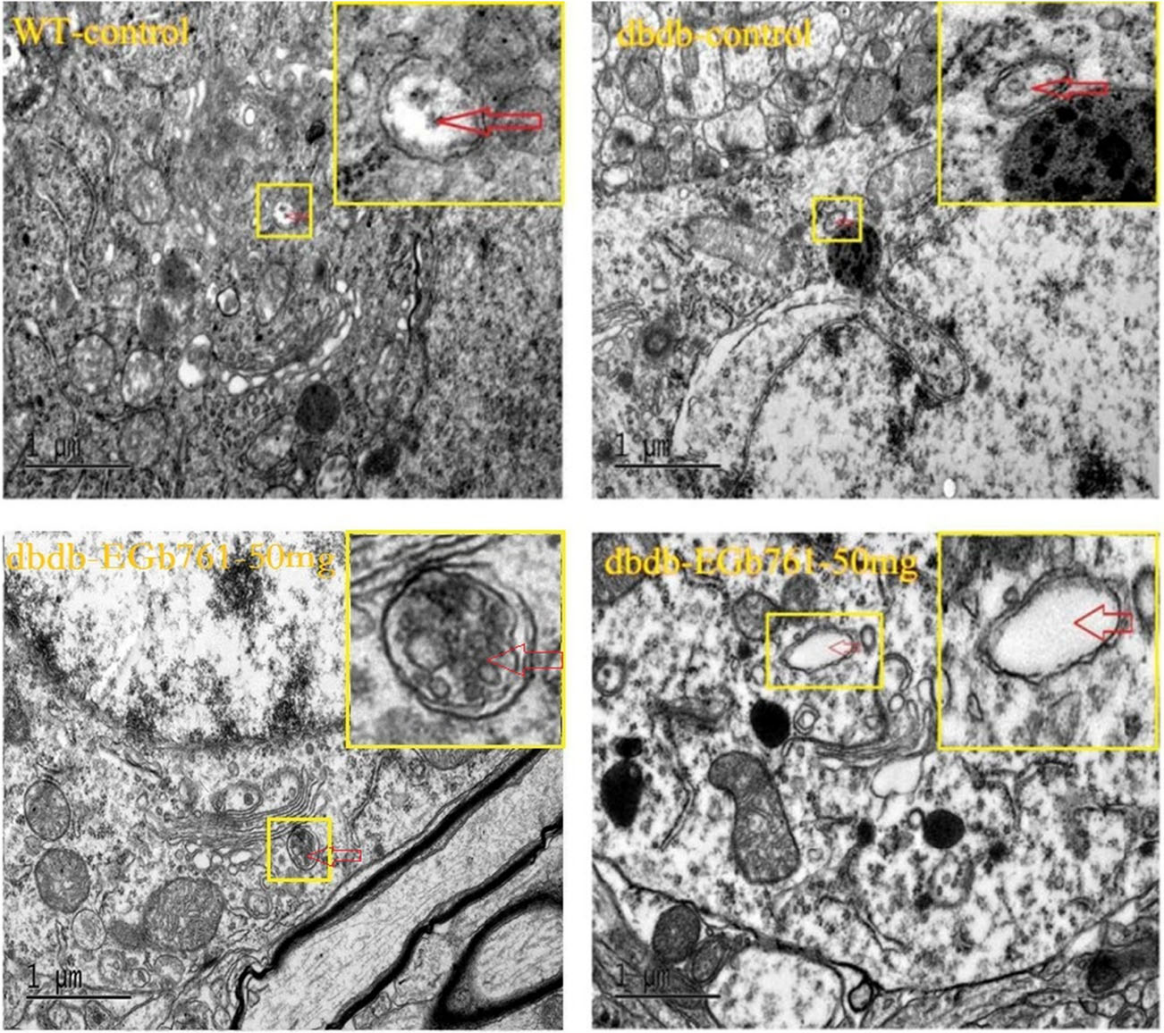
**Ultrastructure alterations in the hippocampus determined using transmission electron microscopy.** There was a trend toward an increased number of autophagosomes (red arrow) in db/db^**−/−**^ mice, but there was no significant difference compared with WT mice. EGb761 did not change obviously the number of autophagosomes. n = 3

TEM was used to assess the ultrastructure of the two sides of the mouse hippocampi to give a clear understanding of the relationship between DM and the brain ultrastructure. As shown in Fig. 7, there was atrend of increased autophagy in the hippocampi of senile db/db^−/−^ mice compared with that in senile WT mice, but did not reach significance. In addition, the number of autophagosomes was not obviously affected by EGb761 (Fig. 7).

## Discussion

With increases in human longevity, a growing number of people are suffering with T2DM (Lefebvre 2005). Both T2DM itself and its complications threaten human health, especially the worst effects occur in the brain. Lacunar infarction is the most common type of CSVD and the brain complication of DM. Since CSVD could lead to cognitive dysfunction, controlling CSVD is researched worldwide. The idea of cognitive dysfunction in DM was proposed as early as 1960 (Miles 1960). According to epidemiological findings, most DM patients had different degrees of cognitive impairment (Awad et al. 2004; Ciechomska et al. 2013). However, the internal causes and pathogenesis are still unclear, and there are lack of effective prevention and treatment measures. Therefore, if CSVD in DM can be caught earlier at the early stages of cognitive dysfunction, it could be possible to delay the memory decline to improve the living quality of DM patients.

The current study used db/db^−/−^ mice with a homogeneous mutation as the T2DM model, and the obese phenotype could be observed at 23 days old. After the mice birthed about two weeks, the plasma insulin and blood glucose levels began to rise. In addition, homogeneous db/db^−/−^ mice exhibited typical symptoms of DM, such as polydipsia, polyuria, increased food intake, and weight loss. In particular, blood glucose levels began to rise significantly and islet cell loss occurred. Most db/db^−/−^ mice have a life span of only 10 months. Therefore, db/db^−/−^ mice with stable blood glucose levels fulfill the requirements for a T2DM model. They are also appropriate for long-term studies assessing whether they develop CSVD and cognitive dysfunction and for studying disease development and mechanisms in depth.

Regarding the autophagy in DM, this pathway of programmed cell death may have variable outcomes (Chen et al. 2014). Under physiological conditions, the levels of autophagy are lower than normal. Under mild stress conditions, autophagy can protect neurons from injury. For example, a slight increase in the level of autophagy can protect the brain cells from death. However, strong stress responses promote cell’s death and even hasten the processes of disease. The current study revealed that the levels of beclin-1 protein were decreased in the hippocampi of db/db^−/−^ mice compared with those in the WT. Therefore, T2DM could reduce the development of autophagy in the hippocampus. Moreover, the levels of autophagy were higher in mice that received EGb761 than in those that did not. However, the levels of LC3 proteins exhibited the opposite trend to beclin-1. The LC3II/I ratio was lower in the db/db^−/−^ groups with EGb761 than in the control group. Autophagosome maturation proceeds with the covalent lipidation of LC3 (Hsu and Shi 2017), and blocking Atg8/LC3 lipidation inhibits the elongation or closure steps of nascent autophagosomes (Sou et al. 2008). Therefore, the current LC3 results suggested that EGb761 could reduce autophagy in T2DM. Beclin-1 plays a role in autophagy via complexes such as the beclin-1-PI3KC3 or beclin-1-Bcl-2 complex, and changing the conditions of the complex could induce or inhibit autophagy. For example, a previous study showed that regulating the beclin-1-Bcl-2 complex by phosphorylation and ubiquitination could block or induce the process of autophagy (Wirth et al. 2013); therefore, the role of beclin-1 in autophagy is complicated. The current TEM results revealed that there was a non-significant trend toward increased autophagy in the hippocampi of senile db/db^−/−^ mice, and the number of autophagosomes was not affected by EGb761. Therefore, we did not clearly define whether EGb761 could enhance autophagy or inhibit T2DM and more studies on this issue are needed in the future.

Previous studies demonstrated that autophagy links inflammation to the oxidative stress responses (Zhou et al. 2011). Although moderate autophagy could prevent the activation of inflammatory bodies, the inflammation would be severe (Hubbard-Lucey et al. 2014). Inflammation is the most significant reaction in many acute or chronic pathological process, and it can alter organizational integrity and the pathways that maintain tissue homeostasis via tissue repair mechanisms. Recently, it was proposed that T2DM is a chronic inflammatory syndrome. The dysfunction of autophagy could induce the inflammation mediated by NF-κB (Fujishima et al. 2011; Crisan TO et al. 2011). In fact, several studies have demonstrated that NF-κB was activated in hyperglycemia and that it had a role in diabetic complications, as reviewed by Patel and Santani 2009 (Patel and Santani 2009). And this hypothesis has also been confirmed in the central nervous system especially in the hypothalamus (Meng and Cai 2011). NF-κB protein, which is expressed widely in mammals, is the core inflammatory molecule. It plays an important role in starting or regulating inflammation. Moreover, NF-κB protein regulates the transcription of many inflammatory factors to enhance inflammatory reactions (Kowalski et al. 2014). Activated NF-κB can enter the nucleus and increase gene expression to enhance immune reactions, inflammatory reactions, multiplication of cells, apoptosis, and aging (Meffert and Baltimore 2005; Vaughan and Jat 2011). The precise regulation of inflammatory reactions is important for repairing tissues, preventing worsening inflammation, and even limiting damage and reducing disease (Goldszmid and Trinchieri 2012). The current study found that not only did autophagy dysfunction occur in the hippocampi of db/db^−/−^ mice, but also that NF-κB levels were higher than in WT mice, and this reminded us that the autophagy dysfunction may cause the inflammatory reactions stronger in hippocampi. This research has provided information which could act as a foundation for future studies of autophagy.

The ginkgo leaves have long been used to produce medicines in China. EGb761, which is extracted from ginkgo leaves, is a strong antioxidant. The active ingredients in EGb761 include flavonoid-glycosides, ginkgolides, and bilobalide, which exert important effects in the body, such as improving microcirculation, increasing the deformability of red blood cells, inhibiting platelet aggregation, and enhancing the oxygen-carrying function of red blood cells. In addition, EGb761 is used to treat cardiocerebral vascular diseases, because it has favorable effects on cerebral circulation, neuronal cell metabolism, and the cholinergic system. It also exerts antioxidant effects, reduces apoptosis, and is neuroprotective against NO- and β-amyloid-induced toxicity (Perry and Howes 2011). A study of T2DM combined with cardiocerebral vascular diseases revealed that EGb761 significantly decreased blood glucose levels, but the mechanism of these effects is still unknown (Lim et al. 2011). Meanwhile, EGb761 can delay cognitive impairment via a possible mechanism involving inhibiting β-amyloid, downregulating the expression of cholesterol acyltransferase, reducing β-amyloid-induced cell death, and upregulating brain-derived neurotrophic factors to protect the neurons from damage (Xiao et al. 2010). Our results of social test suggested that db/db^−/−^ mice had difficulty with novelty recognition, and EGb761 could significantly reduce the preference for unknown subjects in db/db^−/−^ mice and reverse the social preference back to the levels of WT mice. But there were no significant different in WT/db/db^−/−^ group with or without EGb761. It meant that the EGb761 could only improve the social tendancy but not social memory in db/db^−/−^ mice.

To investigate the effects of EGb761 on memory acquisition, the Y-maze task was used to test the spatial recognition memory status. CSVD and cognitive dysfunction were more common in db/db^−/−^ mice than in WT, and low-dose EGb761 could help promote the ability of learning and memory in db/db^−/−^ mice. But there was no significant effect in the higher dose EGb761 group, which with higher error bars has no significance when compared with db/db^−/−^ control. Although the result differed somewhat from our initial expectations, this was the actual experimental result, which might be due to a variety of possible factors including mice individual differences or some systematic errors. Compared with those in WT mice, the nerve function in the hippocampi of db/db^−/−^ was decreased and the connections between the hippocampi and normal brain areas were reduced. In addition, T2DM was shown to affect the functions of the hippocampi via complex signaling pathways which could induce learning and memory dysfunction. The area named CA1 in the hippocampus is most tightly connected to learning and memory, and it is sensitive to hypoxia (Burda et al. 2005). Therefore, we hypothesized that low-dose EGb761 could improve neuronal function in CA1, moderately increase the levels of autophagy, or reduce the inflammatory reactions to protect the vessels from injury. However, high-dose EGb761 had fewer effects on the cognitive function of db/db^−/−^ mice. It may be because that too many db/db^−/−^ mice in the high-dose group died and so the sample size was too small and the system error was large. These results and the mechanism behind them should be confirmed in future studies.

## Conclusions

In conclusion, CSVD and cognitive dysfunction were more common in db/db^−/−^ mice than in WT mice. Autophagy and inflammation might play an important role in the development of CSVD. Low-dose EGb761 could significantly improve the cognitive dysfunction by restoring the function of the hippocampus, improving the autophagy deficit, or downregulating inflammation to protect neurons. Thus, EGb761 could slow down the brain damage and cognitive dysfunction caused by T2DM.

### Fundings

This work was supported by grants from the National Natural Science Foundation, China (No.81170322, No.81571361 and No.81301578), Shanghai Municipal Outstanding Talent Development Foundation (No.2012052), the Taishan Scholars Program (No. tsqn20161070), and Shandong Provincial Natural Science Foundation, China (ZR2014HM069 and ZR2014HM002).
